# Enhanced production of IGF‐I in the lungs of fibroproliferative ARDS patients

**DOI:** 10.14814/phy2.12197

**Published:** 2014-11-04

**Authors:** Graciela Andonegui, Peter M. Krein, Connie Mowat, Ronald Brisebois, Christopher Doig, Francis H. Y. Green, Caroline Léger, Brent W. Winston

**Affiliations:** 1 Department of Critical Care Medicine, Faculty of Medicine, University of Calgary Health Research Innovation Center, Calgary, Alberta, Canada; 2 Immunology Research Group, Faculty of Medicine, University of Calgary Health Research Innovation Center, Calgary, Alberta, Canada; 3 Department of Physiology and Pharmacology, Faculty of Medicine, University of Calgary Health Research Innovation Center, Calgary, Alberta, Canada; 4 Department of Surgery, Faculty of Medicine, University of Calgary Health Research Innovation Center, Calgary, Alberta, Canada; 5 Department of Medicine, Faculty of Medicine, University of Calgary Health Research Innovation Center, Calgary, Alberta, Canada; 6 Department of Pathology and Laboratory Medicine, Faculty of Medicine, University of Calgary Health Research Innovation Center, Calgary, Alberta, Canada; 7 Airway Inflammation Group, Faculty of Medicine, University of Calgary Health Research Innovation Center, Calgary, Alberta, Canada; 8 Department of Biochemistry and Molecular Biology, Faculty of Medicine, University of Calgary Health Research Innovation Center, Calgary, Alberta, Canada

**Keywords:** ARDS, IGF‐I, lung fibroproliferation, PCP‐III

## Abstract

Insulin‐Like Growth Factor I (IGF‐I) has been identified in the lungs of individuals with fibrotic lung diseases. In a previous retrospective study, we showed enhanced IGF‐I immunoreactivity in individuals with fibroproliferative acute respiratory distress syndrome (FP‐ARDS), but we were unable to determine if this correlation was causative. This study was undertaken to prospectively investigate whether IGF‐I expression correlated with the fibroproliferative process and whether IGF‐I was induced and made in the lungs. We measured IGF‐I and procollagen III peptide (PCP‐III) in the epithelial lining fluid (ELF) from controls, early ALI/ARDS patients and FP‐ARDS patients. We also measured IGF‐I mRNA and immunoreactivity from controls and FP‐ARDS patient lung biopsies. We determined the level of lung permeability by measuring albumin and urea levels in ELF and serum. Our data show that IGF‐I is significantly increased in the ELF in FP‐ARDS patients. A significant correlation between IGF‐I and PCP‐III in the ELF of FP‐ARDS patients is found. IGF‐I mRNA is elevated in the FP‐ARDS lung biopsies. Our data suggest that IGF‐I found in the lungs of FP‐ARDS patients results from both increased lung permeability and local production of IGF‐I. The role of IGF‐I in the fibroproliferative process in the lungs has recently been confirmed in an animal model of lung fibroproliferation. This study importantly suggest that IGF‐I protein is made in the lungs of FP‐ARDS patients and correlates with increased levels of ELF PCP‐III, implicating a role for IGF‐I in the fibroproliferative process in humans.

## Abstract (precis)

The role of IGF‐I in the fibroproliferative process in the lungs has recently been confirmed in an animal model of lung fibroproliferation. This study importantly shows that IGF‐I protein is made in the lungs of FP‐ARDS patients and correlates with increased levels of ELF PCP‐III, implicating a role for IGF‐I in the fibroproliferative process in humans.

## Introduction

Acute Respiratory Distress Syndrome (ARDS) is a severe form of acute lung injury whose pathology results from a wide variety of causes, including an increased inflammatory burden (Bhatia and Moochhala 2004). Inflammation leads to diffuse alveolar damage (DAD) with pulmonary edema, increased lung permeability, and reduced gas exchange which may rapidly progress to a fibroproliferative lung process characterized by excessive fibroblast proliferation and collagen deposition (Marshall et al. 2000). Insulin‐like Growth Factor‐I (IGF‐I) is a potent mitogen for fibroblasts and epithelial cells and has been shown to stimulate collagen deposition by fibroblasts in vitro (Goldstein et al. 1989; Olbruck et al. 1998). In vivo, IGF‐I mRNA is detectable in the lungs of mice following bleomycin‐induced pulmonary fibrosis (Maeda et al. 1996), suggesting a role for this mitogen in lung injury, repair, and fibrosis. Unlike normal lungs, the bronchoalveolar lavage fluid (BALF) from patients with fibrotic lung diseases reveals enhanced levels of IGF‐I protein (Rom et al. 1988). Furthermore, in a study of patients with IPF, IGF‐I was localized immunohistochemically to macrophages and epithelial cell populations in the lungs (Uh et al. 1998). Taken together these results suggest enhanced local production of IGF‐I in the fibroproliferative lung, potentially contributing to fibroblast proliferation and collagen synthesis in FP‐ARDS fibrotic lung diseases.

In a retrospective analysis, we showed enhanced IGF‐I immunoreactivity (Krein et al. 2003) and a correlation between IGF‐I and collagen immunoreactivity in lung biopsy specimens from FP‐ARDS patients. This enhanced IGF‐I expression suggests a role for IGF‐I in collagen deposition in the lungs. Indeed, Schnapp et al. (2006) showed increased levels of IGF‐binding protein 3 (IGFBP‐3) and IGF‐I early in ARDS with persistently elevated free IGF‐I for at least 7 days after the onset of ARDS. Furthermore, we recently showed evidence of synergistic fibroproliferation in vivo in an animal model when IGF‐I is coexpressed with TGF‐β1 (Andonegui et al. 2012). While studies of patients with fibrotic lung diseases have shown enhanced IGF‐I protein levels in BALF, the source and levels of IGF‐I protein and its correlation with disease and collagen production in the lungs of individuals with ARDS are not clear.

In this prospective study, we identified individuals with early ALI/ARDS and collected BALF from those who had ALI/ARDS on day one (early ALI/ARDS), ARDS for greater than 5 days (FP‐ARDS or persistent ARDS), as well as from a normal control population. We were able to obtain lung biopsy tissue from two of the 14 FP‐ARDS patients and four of the 11 normal control individuals enrolled in this study. In general lung biopsies are not performed in ARDS, therefore lung biopsy samples are very difficult to get in ARDS patients and as such there were no lung biopsy samples available from patients with early ALI/ARDS and only two biopsy samples from patients with FP‐ARDS. We show expression of IGF‐I protein, collagen I, and collagen III in the lung biopsy specimens obtained from individuals with FP‐ARDS. Importantly, we show enhanced IGF‐I protein and PCP‐III in the ELF from FP‐ARDS patients when compared with the early ALI/ARDS and control populations. The levels of IGF‐I and PCP‐III were significantly correlated, supporting our hypothesis that IGF‐I contributes to collagen deposition in the lungs in FP‐ARDS. Additionally, we show that whole lung IGF‐I mRNA is expressed in the lung biopsy tissue from FP‐ARDS patients compared to controls. This suggests that IGF‐I is induced in the lungs in response to inflammation or injury in FP‐ARDS and likely contributes to the fibroproliferative process.

## Materials and Methods

### Description of the patient cohorts

Patients were eligible for study entry if they had ALI/ARDS for less than 24 h (early ALI/ARDS population) according to the American‐European Consensus Conference definition (Bernard et al. 1994) or if they had ARDS for at least 5 days (FP‐ARDS population), if they had not received steroids within the preceding 4 weeks and were scheduled to undergo bronchoscopy as part of their care. None of the patients enrolled in the early ALI/ARDS or FP‐ARDS groups had known pre‐existing lung disease, evidence of significant clinical improvement over the 12–24 h (early ALI/ARDS) or up to 48 h (FP‐ARDS) prior to lavage, nor had been treated with corticosteroids within 28 days prior to lavage. The control population consisted of individuals who were undergoing bronchoscopy for the evaluation of a pulmonary nodule, to rule out malignant or infectious disease, or prior to wedge resection of pulmonary lesions suspicious for malignancy. This study was approved by the Conjoint Health Research Ethics Board of the University of Calgary and the Calgary Health Region. All clinical measures were taken on study entry at the time of BAL and/or biopsy.

### Bronchoalveolar lavage (BAL) and BAL fluid (BALF) processing

BAL was performed on patients in the intensive care unit or the operating suite. Informed consent was obtained from all patients or legally authorized surrogates involved in this study. In addition, a copy of the consent was given to the patients with the option to opt out after recovery. The patients were sedated and a fiberoptic bronchoscope was wedged in a lung segment. 180 ml of sterile saline in 60 mL aliquots was instilled and retrieved by suction. Fluid returns ranged from 13 to 69% of the 180 mL instilled. Twenty milliliter of the recovered BALF was sent for cell culture and cytology. The surplus was saved for this study. In control cases, BAL was performed on a contra‐lateral lobe to that affected by disease. BALF was placed on ice and filtered through sterile gauze to remove mucus plugs and debris. BALF was centrifuged at 800 × g (15 min, 4°C). Following centrifugation, the BALF supernatant was aliquoted and immediately frozen at −80°C.

### Serum isolation

Four milliliter of blood was drawn by venipuncture, at the same time as the BAL was performed, into a silicone‐coated glass tube. The blood was allowed to coagulate for 1 h at room temperature after which it was centrifuged at 1200 × g for 10 min at 4°C in a swinging bucket centrifuge. The serum was then aliquoted and kept frozen at −80°C until analysis.

### IGF‐I radioimmunoassay (RIA)

BALF specimens were concentrated and reconstituted to five times their original concentration. BALF and serum specimens were prepared for IGF‐I RIA by acid ethanol cryoprecipitation. Samples were analyzed in duplicate using a commercial kit (Nichols Institute, San Juan Capistrano, CA) as described Grogean et al. (1997). Cross reactivity of the assay to IGF‐II was less than 1%. Free (or active) IGF‐I concentrations reported in the ELF were calculated by multiplying the BALF free IGF‐I concentration by the ELF dilution determined using the urea nitrogen method (Rennard et al. 1986).

### Urea nitrogen measurement for ELF protein concentration determination

BALF dilution was calculated by analyzing blood and BALF urea nitrogen using a commercial kit (Sigma, St. Louis, MO). A ratio of BALF urea nitrogen to serum urea nitrogen was used to calculate the dilution of the ELF as previously described Rennard et al. (1986). This dilution factor was applied to all protein calculations for BALF to obtain ELF concentration of the test protein.

### Procollagen III peptide

Procollagen III peptide (PCP‐III) levels were measured in BALF samples using a commercial RIA kit (Orion Diagnostica, Espoo, Finland). PCP‐III concentrations reported in the ELF were calculated by multiplying the BALF PCP‐III concentration by the ELF dilution determined by the urea nitrogen method (Rennard et al. 1986).

### Immunohistochemistry

Biopsy specimens were prepared as previously described Krein et al. (2003). For IGF‐I staining, mouse anti‐human IGF‐I monoclonal antibody (EMD Millipore, Billerica, MA) was used. Slides were counterstained with hematoxylin and were mounted with permount. Collagen I (Fuji Chemicals, Tokaoka, Japan) and Collagen III (Fuji Chemicals, Toyama, Japan) antibodies were used and staining techniques were automated by the Calgary Laboratory Services, Foothills Medical Centre (Calgary, AB). All histology and immunohistochemistry slides were examined by two individuals: P.K. and F.G. (a lung pathologist) blinded to the patient group. After individual assessment, a consensus was reached for each patient on a number of parameters.

### Albumin measurement

Serum albumin was measured using the albumin/bromocresol purple assay (Roche Diagnostics, Laval, PQ) according to the manufacturer's protocol. BALF albumin was measured using the albumin turbidometric Urine and CSF assay (Roche Diagnostics) according to the manufacturer's protocol. Albumin concentrations reported in the ELF were calculated by multiplying the BALF albumin concentration by the ELF dilution determined by the urea nitrogen method (Rennard et al. 1986).

### Real‐time reverse transcription polymerase chain reaction

Of 10 *μ*m lungs tissue sections were heated at 68°C in TRIZOL reagent (Invitrogen Canada Inc, Burlington, ON) for 10 min. Total RNA was then extracted according to the TRIZOL manufacturer's instructions. Real‐time PCR was performed using the Applied Biosystems Prism 7900HT. The following primers/probe sets were used: IGF‐I forward 5′ATGTATTGCGCACCCCTCAA3′, IGF‐I reverse 5′GGGCACGGACAGAGCG3′, and TaqMan Probe containing FAM label 5′ CCTGCCAAGTCAAGC 3′. For CD68, forward 5′CCTCGCCCTGGTGCTTATT3′ CD68 reverse 5′AGGCGGATGGGCGTCT3′ along with FAM labeled TaqMan probe 5′CTTTCTGCATCATCCG3′. For internal control, human 16SrRNA forward 5′GACGGATTACACCTTCCCACTT3′ 16SrRNA reverse 5′TCT TGGCTGATCCATCTGCC3′ and VIC labeled TaqMan probe 5′TGAAAAGGTCAAGGCC3′ were used. Human GAPDH primers with VIC labeled probe were purchased from Applied Biosystems. Fluorescence data, which is relative to mRNA abundance, were plotted versus cycle number and the threshold fluorescence value (ct) set by the applied biosystems software (rRNA for the biopsy specimens) was used to determine mRNA levels for CD68 and IGF‐I. Data are reported as difference in threshold value (dCT), which represents the difference between the cycle number the test mRNA sample passed the threshold versus the cycle number the same control samples (rRNA) passed the threshold. Measures were performed in triplicates. As a result, a more negative number represents more abundant mRNA transcript.

### Statistical analysis

All tests were performed using the STATVIEW software (SAS, Cary, NC). A 95% confidence interval (CI) was used in all the statistical analysis. Statistical differences between BALF and serum proteins and mRNA levels were calculated using the Mann–Whitney *U*‐test. Immunohistochemical correlations were assessed with Spearman's Rank Sum test. Data are represented as mean ± SEM. A *P* < 0.05 was considered significant. For IGF‐I mRNA levels in the lungs, since only 2 FP‐ARDS lung biopsies and four control biopsies were done, the data are presented as mean values without statistical analysis, that is, no error bars are shown in Fig. 5.

## Results

### Clinical parameters

BAL was obtained from 11 control subjects (Table 1), 11 individuals with early ALI/ARDS (Table 2), and 14 individuals with FP‐ARDS (Table 3). There was no significant difference in age or gender between these groups. The control population (Table 1) consisted of individuals who were undergoing bronchoscopy for the evaluation of a pulmonary nodule, to rule out malignant or infectious disease, or prior to wedge resection of pulmonary lesions suspicious for malignancy. In control cases, BAL was performed on a contra‐lateral lobe to that affected by disease. Controls were either never ventilated or did not require mechanical ventilation following thoracic surgery, therefore no blood gas or pulmonary function data are available on the control population. The early ALI/ARDS group had a PaO_2_/FiO_2_ ≤ 250 for less than 24 h (mean 153.7 ± 10.6) and a mean pulmonary compliance of 36.3 (±2.7) mL/cm H_2_O (Table 2). The FP‐ARDS group had clinically defined ARDS for an average of 10.9 (±1.1) days prior to BAL or biopsy, were ventilated for 11.4 (±1.7) days, had a PaO_2_/FiO_2_ ratio of 140.2 (±12.6) and a mean pulmonary compliance of 27 (±1.8) mL/cm H_2_O (Table 3).

**Table 1.** Clinical characteristics of prospective control population. BAL was performed on each of the control patients. BAL samples were taken from the indicated bronchopulmonary segment

Patient	Sex	Age	BAL Lobe	Underlying condition
1	M	49	RML	Noncaseating Granuloma
2	F	67	RML	Lung Cancer
3	M	71	LUL	Metatastic Colon Cancer
4	F	39	LLL	Hamartoma
5	M	67	LUL	Lung Cancer
6	F	49	LLL	Lung Cancer
7	F	73	RML	Lung Cancer
8	M	22	RML	Recurrent Pneumothorax
9	M	63	RUL	Hamartoma
10	M	27	RML	Hemoptysis
11	M	53	LING	Lung Cancer

LUL, left upper lobe; LLL, left lower lobe; Ling, lingula; RML, right middle lobe; RUL, right upper lobe.

**Table 2.** Clinical characteristics of prospective early ALI/ARDS population. BAL was performed on each of the early ALI/ARDS patients. Each patient had ARDS for ≤ 24 h prior to BAL sampling. BAL samples were taken from the indicated bronchopulmonary segment on day 1 of study entry

Patient	Sex	Age	BAL Lobe	Days with ALI/ARDS	PaO_2_/FiO_2_	Compliance	Underlying condition
1	F	48	RML	1	129	45	Multiple Trauma
2	M	69	RLL	1	164	32.5	Post‐op Abdominal Surgery, Sepsis
3	F	56	RML	1	138	40	CVA, SAH, Possible Aspiration
4	M	67	LING	1	182	40	Pneumonia, Sepsis – Culture Negative
5	M	18	RML	1	175	37.4	Trauma, Massive Transfusion
6	F	18	LING	1	170	27	Aspiration Syndrome
7	M	53	LING	1	110	42	Ischemic Gut, Sepsis
8	M	62	RML	1	94	54	Atypical (Possibly Viral) Pneumonia
9	F	41	RML	1	220	26	Sepsis
10	F	43	RML	1	162	29	Multiple Trauma
11	F	72	RLL	1	147	27	Small Bowel Obstruction, Sepsis

Ling, lingula; RML, right middle lobe; RLL, right lower lobe; SAH, subarachnoid hemorrhage.

Days with ALI/ARDS reflects the days after the onset of ALI/ARDS when that BAL was performed. Immediately prior to bronchoscopy and BAL, a blood gas was measured to calculate PaO_2_/FiO_2_ and compliance measurements (mL/cm H_2_O) were undertaken.

**Table 3.** Clinical characteristics of prospective FP‐ARDS population. BAL was performed on each of the FP‐ARDS patients. Each patient had ARDS for >5 days prior to BAL sampling. BAL samples were taken from the indicated bronchopulmonary segment on the day of study entry

Patient	Sex	Age	BAL Lobe	Days ARDS	PaO_2_/FiO_2_	Compliance	Underling condition
1	M	60	LLL	7	94	30	Coronary Artery Disease, Hypertension, Gout
2	M	28	LUL	6	211	31	Myocardial Infarction
3	M	27	LUL	7	60	24	Tibial Fracture, Fat Emboli
4	M	44	LUL	21	195	41	Pneumonia, Acute Tubular Necrosis
5	M	16	LLL	12	143	24	Multiple Trauma, Fat Emboli
6	M	29	LUL	9	150	35	Acute Renal Failure
7	M	49	LLL	12	112	18	Lung Cancer
8	F	42	LLL	15	100	30	ASA Overdose, Pulmonary Hypertension
9	M	68	LLL	9	170	25	Pulmonary Hypertension, Type II Diabetes
10	M	62	RML	10	81	30	Meningitis, Type I Diabetes, EtOH, Hepatitis B, Coronary Artery Disease
11	M	60	LING	10	153	n/a	Sepsis, Perforated Duodenal Ulcer
12	M	26	LUL	11	143	22	Sepsis
13	M	34	LUL	16	142	20	Multiple Trauma, Liver and Spleen Lacerations
14	F	73	RML	7	209	21	Lung Cancer, Lobectomy

LUL, left upper lobe; LLL, left lower lobe; Ling, lingula; RML, right middle lobe; n/a, data not recorded; EtOH, alcohol abuse.

Days ARDS reflects the days after the onset of ARDS when that BAL ± biopsy was performed and the timing of measurement of PaO_2_/FiO_2_ and compliance measurements (mL/cm H_2_O).

### IGF‐I protein (Free or Active)

**Figure 1.**
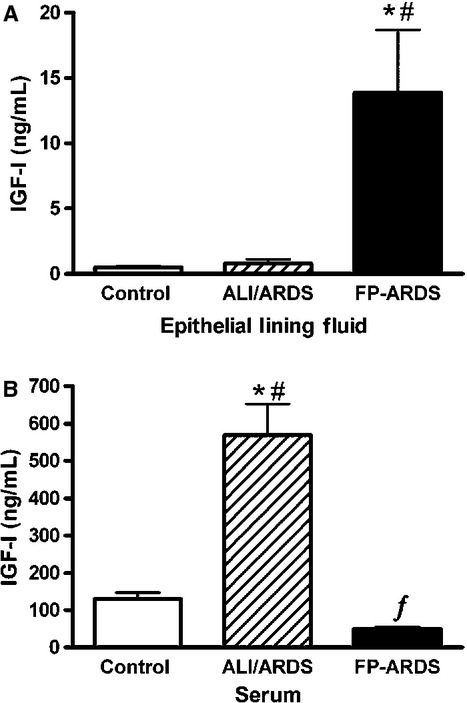
Free (Active) IGF‐I Protein Levels in ELF and Serum. Free IGF‐I protein levels were measured in BALF and serum by RIA. ELF concentration was calculated using the urea method to correct for BAL dilution as described in Materials and Methods. (A) Free IGF‐I protein levels are elevated in FP‐ARDS ELF (*n* = 14) versus control individuals (*n* = 11), *P < 0.05, 95% CI and versus early ALI/ARDS ELF (*n* = 11), ^#^*P* < 0.05, 95% CI. (B) In early ALI/ARDS serum (*n* = 11) free IGF‐I protein levels increase significantly compared to control (*n* = 11), *P < 0.0001 and FP‐ARDS patients (*n* = 14) ^#^*P* < 0.0001. In FP‐ARDS patients free IGF‐I serum levels are significantly lower than control individuals ^ƒ^*P* < 0.0001, 95% CI.

Free IGF‐1 levels were determined in the ELF and serum of control, ALI/ARDS and FP‐ARDS patients. Figure 1A shows a significant increase in free IGF‐I levels in the ELF of FP‐ARDS patients (13.9 ± 4.8 ng/mL) compared to early ALI/ARDS (0.8 ± 0.3 ng/mL) and control (0.5 ± 0.1 ng/mL) groups. In several control BAL specimens the level of free IGF‐I protein was below the detection limit of the assay (0.06 ng/mL). For these samples, a value of 0.06 ng/mL was recorded for IGF‐I concentration and used to calculate the mean ELF concentration of free IGF‐I. These results are consistent with our previous findings where IGF‐I immunoreactivity was enhanced in FP‐ARDS patients (Krein et al. 2003). On the other hand, serum free IGF‐I levels were elevated in the early ALI/ARDS group (569.2 ± 83.5 ng/mL) compared to the levels recovered in the controls (129.5 ± 17.8 ng/mL) and FP‐ARDS group (50.6 ± 4.8 ng/mL) (Fig. 1B). These results are consistent with previous reports of reduced IGF‐I production from the liver during sustained inflammation, such as occurring during FP‐ARDS (ARDS ≥ 5 days) (Thissen and Verniers 1997).

### PCP‐III protein BAL

**Figure 2.**
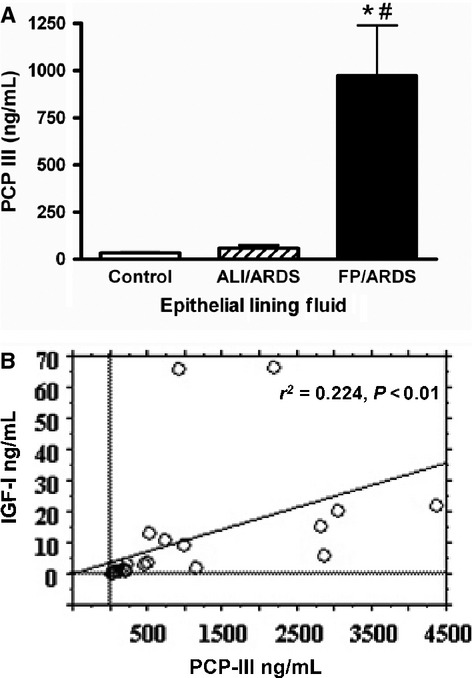
PCP‐III Protein in ELF. PCP‐III protein levels measured in BALF by RIA. ELF concentration corrected for dilution by the urea method as described in Materials and Methods. (A) PCP‐III protein levels are increased in the ELF of FP‐ARDS individuals (*n* = 14) versus control individuals (*n* = 11), *P < 0.01, 95% CI and versus early ALI/ARDS individuals (*n* = 9), ^#^*P* < 0.05, 95% CI. (B) A significant positive relationship exists between IGF‐I and PCP‐III levels in the ELF (*P* < 0.01, 95% CI) of FP‐ARDS. IGF‐I and PCP‐III levels were analyzed by Spearman Rank Correlation analysis as described in Materials and Methods.

IGF‐I has been shown to induce collagen production in fibroblasts in vitro (Goldstein et al. 1989; Olbruck et al. 1998), suggesting one of the consequences of enhanced IGF‐I protein in the lungs of FP‐ARDS patients may be increased collagen deposition in this organ. Therefore, we measured PCP‐III, a byproduct of collagen III processing as a surrogate for collagen deposition in the lungs. Figure 2A shows increased PCP‐III levels in the ELF from FP‐ARDS patients compared to both early ALI/ARDS and controls. Importantly, the protein levels of IGF‐I and PCP‐III in the ELF of FP‐ARDS are significantly correlated versus control individuals (*P* < 0.01) (Fig. 2B). These results further support the hypothesis that IGF‐I may induce or contribute to collagen production and deposition in the lungs of FP‐ARDS patients.

### IGF‐I and collagen immunohistochemistry

**Figure 3.**
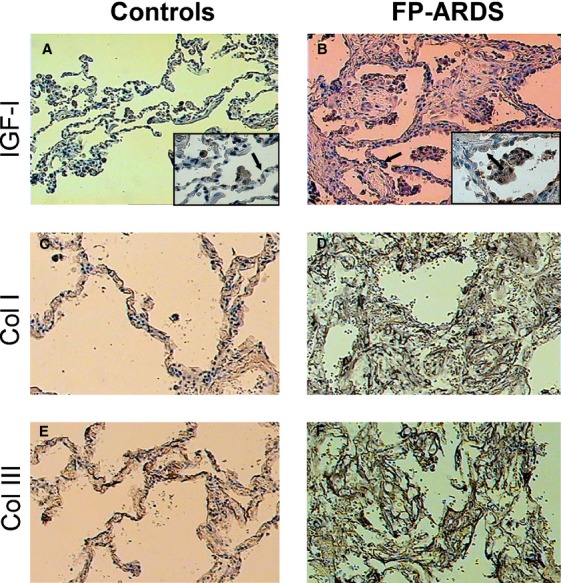
IGF‐I, Collagen I and Collagen III Immunohistochemistry of Lung Biopsies. (A) Representative staining for IGF‐I immunoreactivity in lung biopsy specimen of a control individual at low magnification (100×) showing normal alveolar architecture. There is minimal IGF‐I positive immunostaining in the control lungs as evidenced by the positive immunostaining in a cluster of alveolar macrophages. High magnification inset (400×) shows the positive immunoreactivity of the alveolar macrophages as well as a minimal amount of linear staining of the alveolar type I cells (arrow). (B) Representative staining for IGF‐I in lung biopsy specimen of a FP‐ARDS patient. Low magnification (100×) shows thickening and fibrosis of the interstitium with chronic inflammatory cell infiltrates. Numerous strongly stained macrophages are seen in the alveolar lumen as well as scattered epithelial cells (arrow). Interstitial staining is also present but less strong. High power inset (400×) shows strong immunoreactivity of macrophage cells. (C) Representative collagen I immunostaining in control lung section and (D) fibroproliferative ARDS lung section. Of note, there is little collagen I staining in control lung sections but significant collagen I immunostaining in the interstitium of FP‐ARDS lung sections. (E) Representative collagen III immunostaining in control lung section and (F) FP‐ARDS lung section. Noteworthy, there is little collagen III staining in control lung sections but significant collagen III immunostaining in the interstitium in FP‐ARDS lung sections.

The presence of IGF‐I was confirmed immunohistochemically in lung biopsy samples from two FP‐ARDS and four control individuals enrolled in this study. Control lung sections showed normal lung architecture with minimal alveolar structure distortion, little cellular infiltration, or thickening of the interstitium (Fig. 3A), whereas the lung sections from FP‐ARDS patients showed enhanced mononuclear infiltrates, loss of alveolar structure, and an increase in interstitial mass (Fig. 3B). IGF‐I immunoreactivity was seen in alveolar macrophages, epithelial cells lining the airways as well as in a variety of interstitial cells in control lungs (Fig. 3A). There was increased total staining for IGF‐I in the two FP‐ARDS biopsy samples compared to the four control biopsy specimens (Fig. 3B) as assessed by two different observers. These findings are in keeping with those previously reported Krein et al. (2003). Additionally, the lung biopsy specimens from the FP‐ARDS individuals showed enhanced collagen I (Fig. 3 C and D), and collagen III immunoreactivity (Fig. 3 E and F) compared to controls, which is in agreement with the results observed in the BALF of these patients. This confirms that the FP‐ARDS patients entered into this study with a clinical diagnosis of FP‐ARDS have clear ongoing fibroproliferation in their lungs.

### Is the lung the source of IGF‐I in FP‐ARDS?

**Figure 4.**
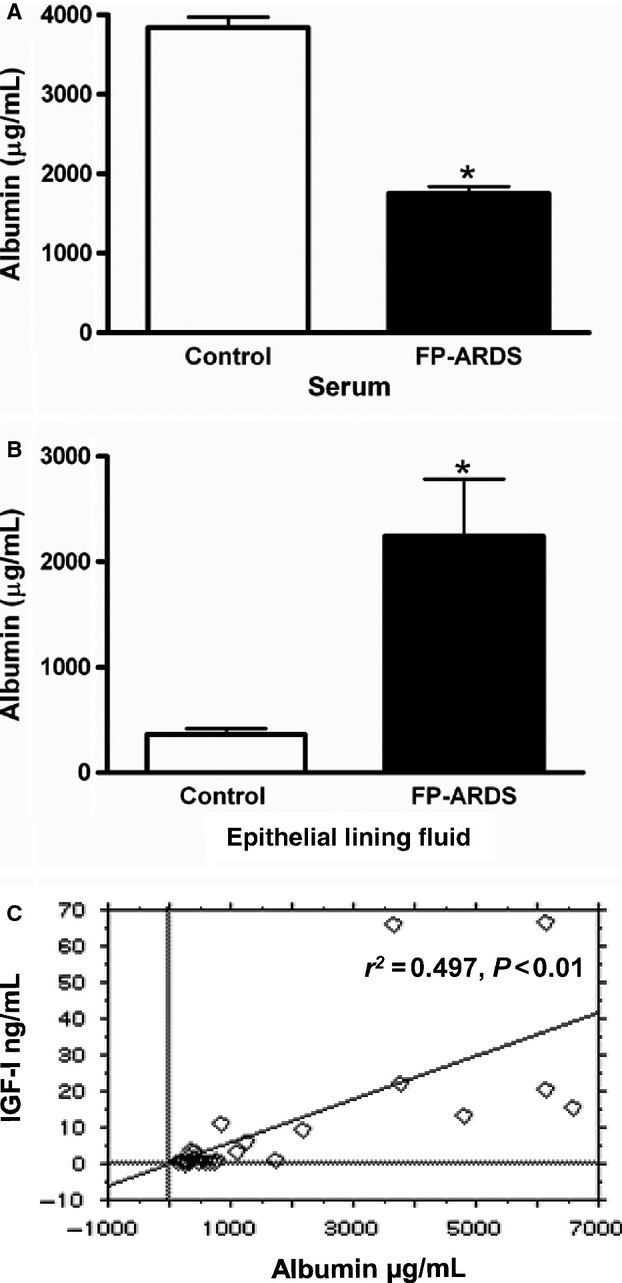
Albumin Levels in ELF and Serum. (A) Serum albumin levels are decreased in FP‐ARDS individuals (*n* = 12) versus controls (*n* = 10), *P < 0.01, 95% CI. (B) ELF albumin levels were calculated by measuring BALF albumin levels and normalizing BALF dilution by the urea method as described in the Materials and Methods. Results show increased albumin in FP‐ARDS (*n* = 14) versus control (*n* = 11), *P < 0.01, 95% CI. (C) A significant positive relationship exists between free IGF‐I and albumin levels in the ELF of the prospective BAL populations, *P* < 0.01, 95% CI using Spearman Rank Correlation.

**Figure 5.**
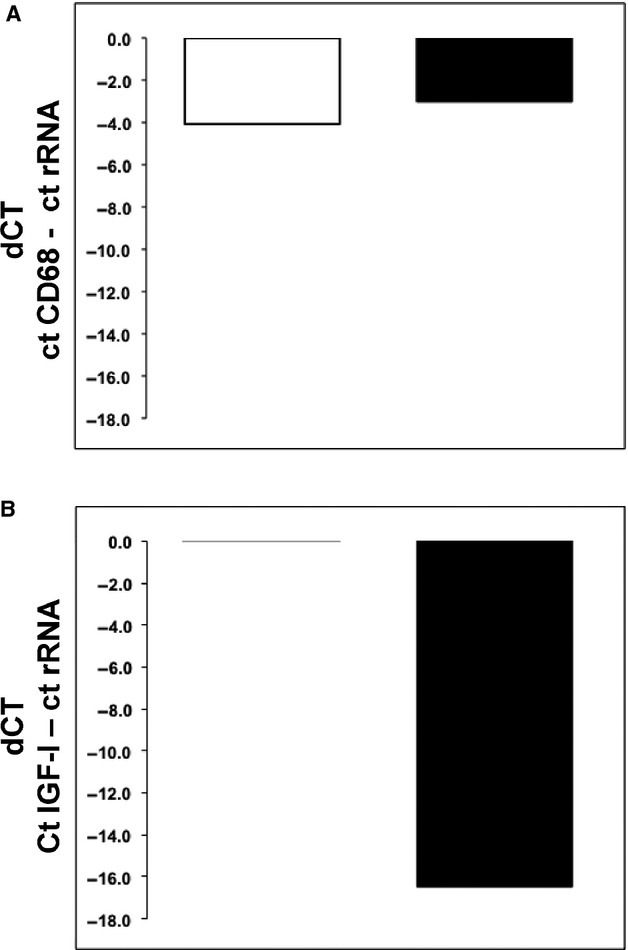
Real‐Time qRT‐PCR of Lung Biopsy Specimens. mRNA levels measured in control (*n* = 4 in triplicate) and FP‐ARDS (*n* = 2 in triplicate) lung biopsy specimens. Threshold values (CT) were subtracted from rRNA threshold value giving dCT for each patient. Displayed are average dCT values. A more negative number indicates higher mRNA expression. (A) CD68 mRNA quantified from lung biopsy specimens in control and FP‐ARDS biopsies. (B) IGF‐I mRNA quantified from control and FP‐ARDS lung biopsy specimens. IGF‐I mRNA is below the level of detection in control lung biopsy specimens. Of note, there are increased IGF‐I mRNA levels in the FP‐ARDS patients compared to controls. This difference is not seen when CD68 mRNA (a macrophage marker) is measured in control and FP‐ARDS lung biopsy specimens.

As we detected high levels of IGF‐I in the serum of the early ALI/ARDS population, we sought to determine whether the lung IGF‐I protein increase in the FP‐ARDS population was the result of local production or the result of leakage of IGF‐I from the serum through increased lung permeability. First, we measured the levels of albumin in the ELF and serum samples from our prospective patient populations, as previously described Rennard et al. (1986). As shown in Fig. 4A, albumin levels are decreased in the serum of our FP‐ARDS patients whereas albumin levels normalized for BALF dilution are significantly increased in the ELF of FP‐ARDS patients compared to controls (Fig. 4B). These results suggest a significantly increased permeability of the epithelial:endothelial barrier in the lungs of FP‐ARDS patients. Figure 4C shows a significant positive relationship between free IGF‐I and albumin in the ELF (*P* < 0.01) of FP‐ARDS patients which suggests that the elevated IGF‐I protein in the ELF could in fact be derived from the serum, and arrives in the lung via vascular leak. However, this is not the case as when albumin levels were used to account for serum leak in the patient lungs, the FP‐ARDS population still had increased IGF‐I in the ELF compared to controls. As shown in Fig. 4B there is a 6.2‐fold increase in albumin leak when one compares ELF albumin from FP‐ARDS patients versus controls. On the other hand, there is a 24‐fold increase in the IGF‐I ratio when one compares the ELF IGF‐I from FP‐ARDS patients vs. controls (Fig. 1A), strongly suggesting that serum leak as measured by the increase albumin leak ratio, cannot account for all of the IGF‐I in the lungs of FP‐ARDS patients. In support of these data, each of the two FP‐ARDS lung biopsy specimens showed a high level of IGF‐I mRNA, whereas IGF‐I mRNA was not detected in any of the control lungs biopsies (Fig. 5B). These results strongly suggest that there is local production of IGF‐I in the lungs of FP‐ARDS patients.

### What is the cellular source of IGF‐I in FP‐ARDS?

In a previous study, we were able to show increased IGF‐I immunoreactivity in lung macrophages on FP‐ARDS lung biopsy specimens compared to controls (Krein and Winston 2002). In this study, we quantitatively amplified rRNA, the macrophage marker CD68 and IGF‐I mRNA from two FP‐ARDS lung biopsy specimens (Fig. 5). Interestingly, in our four control patients, we detected rRNA and CD68 mRNA at similar levels as that found in the FP‐ARDS group (Fig. 5A), but IGF‐I mRNA was not detected in controls (Fig. 5B). In contrast, IGF‐I mRNA was elevated in the FP‐ARDS lungs (Fig. 5B). The equivalent rRNA and CD68 levels suggest that mRNA was similarly intact in the control and FP‐ARDS specimens, and that the level of macrophage infiltration was similar between these two groups. This would suggest that lung macrophages are not an appreciable source of IGF‐I in FP‐ARDS patients. Furthermore, we found that the IGF‐I mRNA present in the alveolar macrophages isolated from the BALF of our FP‐ARDS patients (data not shown) was not elevated when compared with the levels recovered in alveolar macrophages isolated from the control population, confirming our suspicion. The alveolar macrophages do not appear to be an appreciable source of increased IGF‐I message in FP‐ARDS. As the immunoreactivity for IGF‐I was increased in several cell types in the lung, it suggests that cells other than macrophages likely account for the increased IGF‐I message in the lungs of FP‐ARDS patients such as type I and alveolar epithelial cells as seen in Fig. 3.

## Discussion

In this prospective study we extend the findings of our previous retrospective study, showing that IGF‐I in the lungs of FP‐ARDS patients is significantly higher than in early ALI/ARDS and controls (Krein et al. 2003). Indeed, free IGF‐I protein is significantly elevated in the ELF of FP‐ARDS patients as well as in lung biopsy specimens when compared with control individuals. Of note, free IGF‐I protein is not increased in the serum of FP‐ARDS patients. Furthermore, we show increased IGF‐I protein and collagen I and III deposition in the lungs of FP‐ARDS patients suggesting that IGF‐I may contribute to the fibroproliferative process in FP‐ARDS. Finally, we show that total lung IGF‐I mRNA is expressed in FP‐ARDS compared to control lungs suggesting that IGF‐I is being produced in the lungs of FP‐ARDS patients. The elevated lung IGF‐I levels seen in the FP‐ARDS population is likely a combination of local induction in addition to IGF‐I moving into the lungs due to the enhanced permeability seen in the damaged lungs of FP‐ARDS patients. Interestingly, in early ALI/ARDS, the ELF free IGF‐I protein and collagen deposition marker levels (PCP‐III) are low, whereas in the serum free IGF‐I protein is increased versus control and FP‐ARDS. The strong correlation between IGF‐I and PCP‐III in FP‐ARDS strengthens the hypothesis that IGF‐I is an important profibrotic agent in the lungs of FP‐ARDS patients.

There are two relatively recent papers that examine IGF‐I and IGFBP3 levels in ARDS. In the first one, Schnapp et al. (2006) have previously shown that although the total IGF‐I protein levels may decrease by 7 days, the free IGF‐I protein concentration in the lungs is elevated at that time in the ARDS patients compared to controls. We confirmed this observation; although the level of free IGF‐I is not significantly elevated in the ELF of our early ALI/ARDS group (≤24 h after the onset of ALI/ARDS) it is significantly increased in our FP‐ARDS group (≥5 days of onset of ARDS). In the second one, Ahasic et al. (2012) have recently shown that lower plasma levels of IGF‐I and IGFBP3 are associated with mortality among the ARDS patients. Interestingly, our data show that lower levels of serum free IGF‐I levels are observed in FP‐ARDS patients. It is clear that FP‐ARDS patients have a higher mortality relative to early ARDS (reviewed in Burnham et al. 2014).

There is apparent discrepancy between the alveolar macrophage data where we see both control and FP‐ARDS producing IGF‐I mRNA and the lung biopsy specimens where only FP‐ARDS biopsy specimens contain IGF‐I mRNA may be explained by the fact that macrophages may be a relatively minor source of lung IGF‐I at all times, whereas in FP‐ARDS a variety of other cells (including epithelial cells) may actively produce IGF‐I. Additionally, macrophages may produce IGF‐I in the airspaces early in disease or in a homeostatic manner, whereas late in disease, when the pathology is primarily an interstitial fibroproliferative process, the IGF‐I production may have shifted to the interstitial or epithelial cells not recovered in the BALF. While we present evidence that IGF‐I mRNA is induced locally in the lung, we cannot determine what proportion of the IGF‐I protein found in the lung is a result of local production versus that which has arrived from increased lung permeability. Nor can we be certain of what cell type is responsible for the increase in IGF‐I mRNA production though, based on the fact the BAL alveolar macrophages from FP‐ARDS patients contain no more IGF‐I mRNA than BAL macrophages from controls, it is unlikely to be alveolar macrophages contributing significantly to the increased IGF‐I mRNA in FP‐ARDS patients.

Regardless of the source, IGF‐I is likely contributing to the fibroproliferative process in the lung, including collagen deposition, as judged by the association between IGF‐I and PCP‐III. Indeed, in the ELF of the ALI/ARDS group, the levels of IGF‐I and PCP‐III are both not significantly different from controls, whereas in the patients who have progressed to fibroproliferative ARDS, these levels are both increased several fold compared to controls.

The data presented in this study show that there are elevated levels of free or active IGF‐I in FP‐ARDS. This is highly relevant because in a recently published manuscript (Andonegui et al. 2012), using an in vivo animal model, we have shown that IGF‐I plays a synergistic fibroproliferative role in lungs in the presence of TGF‐β1 Importantly, others have shown that patients with ALI/ARDS have elevated active TGF‐β1 levels in their lungs (Budinger et al. 2005). Further support for the role that IGF‐I is playing in lung fibroproliferation comes from Choi et al. (2009), where use of an IGF‐IR tyrosine kinase receptor blocker in a bleomycin model of lung fibrosis in mice resulted in less fibrosis when compared with controls.

In this study, we provide evidence of enhanced IGF‐I protein in the lungs of individuals with FP‐ARDS and provide further support for a role of IGF‐I in the fibroproliferative process by showing the induction and presence of IGF‐I in concert with enhanced levels of PCP III (a precursor of collagen III deposition) and collagen in the lungs. This supports a role for IGF‐I in the progression of early ARDS to FP‐ARDS. Importantly, we provide evidence that IGF‐I is present in the lungs both from leakage through the endothelial:epithelial barrier as well as local induction/production in the lungs.

We acknowledge some limitations to this study. This is a prospective observational study and thus no blinding was performed. However, data analysis, where possible, were carried out in a blinded fashion. We recognize the limitation of having only 2 FP‐ARDS lung biopsies to examine IGF‐I mRNA induction. Despite extensive efforts to obtain more patient biopsy material, and despite ethics approval to do so, many clinicians felt biopsies were not necessary for the diagnosis and would not obtain biopsies of FP‐ARDS patients. Because of this, we present data showing only the average of two biopsy patient IGF‐I mRNA levels. Thus, our small sample size only allowed us to point out interesting observations, which will need to be confirmed in a larger study ± blinding. Nonetheless, as our data shows minimal variation between samples, and the biopsy results are completely consistent with the immunohistochemistry data presented in our previous retrospective study (Krein et al. 2003) we are confident of the validity of these results. In addition, our data are in agreement with the finding of others (Schnapp et al. 2006).

## Acknowledgments

In memory of Connie Mowat who passed away during the preparation of this manuscript. Peter Krein was supported by an Alberta Heritage Foundation for Medical Research (AHFMR) studentship. Brent Winston was supported by an AHFMR Scholarship. We thank Drs. Alex Izakson and Teik‐How Lim for their intellectual contribution. We thank Drs. Paul Boucher, Sean McFadden, Gary Gelfand, Jeffory Mellor, and Chris Mody from the Foothills Medical Centre for assistance in obtaining BALF samples; Julie Burgess and Dr. Cliff Rosen from the Maine Center for Osteoporosis Research Laboratory for measuring the IGF‐I protein in the BALF and serum samples; and Susan Hui of the Calgary Laboratory Services, Histopathology Division for immunohistochemical staining assistance. Finally, we thank The Snyder Translational Laboratory in Critical Care Medicine for use of their facilities.

## Conflict of Interest

None declared.
